# Bioavailability of Plant-Derived Antioxidants

**DOI:** 10.3390/antiox2040309

**Published:** 2013-11-05

**Authors:** Ehab A. Abourashed

**Affiliations:** Department of Pharmaceutical Sciences, College of Pharmacy, Chicago State University, Chicago, IL 60628, USA; E-Mail: eabouras@csu.edu; Tel.: +1-773-821-2159; Fax: +1-773-821-2595

**Keywords:** plant antioxidants, antioxidant natural products, bioavailability, carotenoids, polyphenols, organosulfur compounds, glucosinolates

## Abstract

Natural products with antioxidant properties have been extensively utilized in the pharmaceutical and food industry and have also been very popular as health-promoting herbal products. This review provides a summary of the literature published around the first decade of the 21st century regarding the oral bioavailability of carotenoids, polyphenols and sulfur compounds as the three major classes of plant-derived antioxidants. The reviewed original research includes more than 40 compounds belonging to the above mentioned classes of natural antioxidants. In addition, related reviews published during the same period have been cited. A brief introduction to general bioavailability-related definitions, procedures and considerations is also included.

## 1. Introduction

The search for new antioxidant compounds is an ongoing area of drug discovery, and the plant kingdom has been generous in providing hundreds of diverse natural products with such activity. Polyphenols, carotenoids, glucosinolates and different vitamins represent some of the major classes to which natural antioxidants belong. One of the main goals of drug discovery (antioxidants included) is to identify lead compounds with enhanced activities. This is often achieved via *in vitro* evaluation as the first step [1]. It is not uncommon, however, that the identified activity is lost once the same compound transitions to the *in vivo* evaluation phase. An obvious reason for the “loss” of activity is the lack of pharmacokinetic optimization or compatibility [1]. One of the main factors that influences pharmacokinetics is the tissue bioavailability of the tested entity. An abbreviated definition of bioavailability is “the fraction of administered drug that can reach plasma and body tissues in an unchanged form” [2]. The impact of bioavailability in drug discovery and development is even more pronounced, with products intended for oral use, whereby gastro-intestinal (GI) absorption constitutes the primary barrier between an active ingredient and systemic circulation. Thus, bioavailability should also be considered when the efficacy of herbal dietary supplements is evaluated in animal models and/or human clinical trials. The goal of this review is to focus on oral bioavailability as a major pharmacokinetic aspect of antioxidant natural products discovery and development. In this respect, factors affecting oral absorption of active compounds will be addressed. *In vitro* and *in vivo* methods currently utilized for the evaluation of oral bioavailability will be summarized, and a survey of related bioavailability studies for members of different classes of antioxidants will be presented.

## 2. Factors Affecting Oral Absorption of Xenobiotics

**Figure 1:**
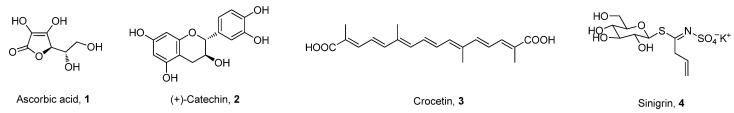
Representative orally bioavailable natural antioxidants and antioxidant modulators with diverse chemical structures.

Biological membranes, including the GI wall, act as lipid barriers against drug absorption. The chemical (e.g., pH) and biological environment (e.g., microbial flora) inside the GI tract also have a significant influence on drug absorption [2]. A drug that can exist in a stable form to survive the GI environment and that has optimum physico-chemical properties to penetrate the GI wall is most likely to possess acceptable oral bioavailability. The optimum properties for GI absorption of drug molecules have been defined by Lipinski’s rule [3]. According to this predictive model, a molecule is expected to display optimum GI absorption if it has a molecular weight of 500 daltons or less, no more than five hydrogen bond donors, no more than 10 hydrogen bond acceptors and a calculated partition coefficient (LogP) that is no more than five. Molecules that are absorbed by specific transporters are an exception to this rule. The presence of adjuvants, e.g., food or drugs, can also have a significant effect on the bioavailability of certain compounds by influencing their bioaccessibility (GI availability after digestion) and transport across the apical and basolateral membranes of the GI tract [2]. Ascorbic acid (**1**), catechin (**2**), crocetin (**3**) and sinigrin (**4**) (Figure 1) are examples of chemically unrelated natural products that meet Lipinski’s requirements for acceptable oral bioavailability, and as such, they are pharmacologically active *in vivo*.

## 3. Evaluation of Oral Bioavailability

**Figure 2:**
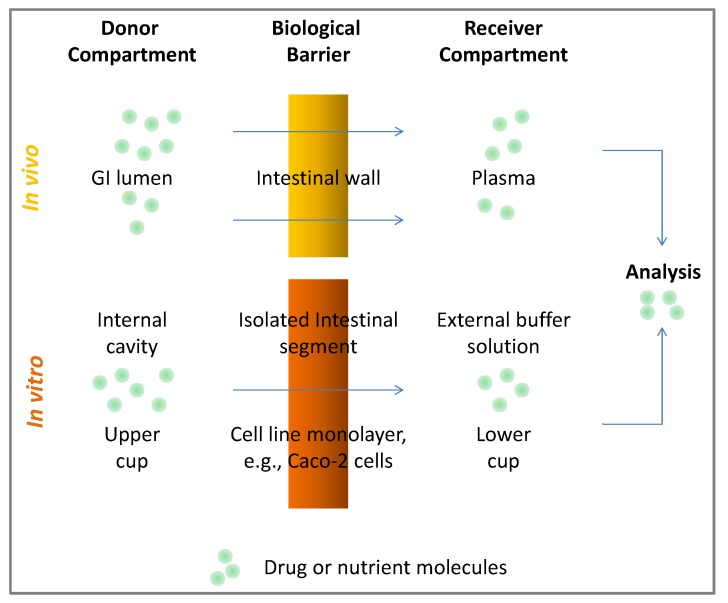
General components of bioavailability evaluation procedures. GI, gastro-intestinal.

Any experimental approach geared towards the investigation of intestinal absorption of bioactive molecules incorporates: (i) a barrier membrane separating the donor compartment, *i.e.*, GI lumen, from the receiver compartment, *i.e.*, blood circulation, through which the molecule has to pass; and (ii) an analytical method of appropriate selectivity and sensitivity to quantify the amount of drug transported across the membrane. Similar to bioactivity screening, evaluation of bioavailability can be conducted *in vitro*, *in vivo* or both. As to be expected, *in vivo* evaluation of oral bioavailability is usually conducted in mammalian models (e.g., mice, rats, dogs and monkeys), non-mammalian models (e.g., arthropods) and human clinical trials. Although *in vivo* evaluation is more relevant, *in vitro* (*ex vivo*) models of oral bioavailability are also available and rely on using isolated organs or cultured cell monolayers as the barrier membrane. In the former case, isolated intestinal preparations of various mammalian origins are commonly used, while human colon cancer type 2 (Caco-2) cell lines are commonly used for the latter [2]. Due to a number of advantages, the Caco-2 cell lines have been successfully utilized as a monolayer barrier that resembles the intestinal epithelial lining [4]. Other cell lines, such as Madin-Darby canine kidney cells (MDCK), rat fetus intestinal cells and pig kidney epithelial cells (LLC-PK1), are also known, but are less commonly used, due to certain limitations compared to Caco-2 cells [2]. Suspended mammalian intestinal brush border membrane vesicles (BBMV) have also been utilized as *in vitro* models for studying transport across cell membranes [5]. The most commonly used analytical method for monitoring drug transport across the gastrointestinal barrier is HPLC with UV or MS (LC-MS) detection. Mass spectrometric detection is significantly more specific and sensitive than UV detection and is, thus, more appropriate to use with *in vivo* studies [6]. Both sensitivity and selectivity are significantly enhanced when tandem mass detectors are used (LC-MS-MS) [7]. The general approach towards the evaluation of oral bioavailability is depicted in Figure 2.

## 4. Bioactivity and Bioavailability: Are They Closely Matched?

Reviewing the current literature for the discovery/evaluation of plant-derived antioxidants often shows statements, such as “little is known about *in vivo* activity”, “*in vivo* evaluation is thus warranted” and “further studies are needed to establish the bioavailability of tested compound(s)”. All such statements deliver the message that many bioavailability-related questions need to be answered and that *in vitro* data are only as good as what they are intended for, *i.e.*, preliminary evaluation of activity [1]. With that said, the literature also reveals that the major classes of plant-derived antioxidants have been subject to numerous studies addressing their oral bioavailability characteristics. During the past 15 years, numerous reviews and research papers have been published about the bioavailability of individual classes of natural antioxidants. The following section will cite published reviews and will provide a summary of the studies conducted to evaluate the oral bioavailability of plant carotenoids/apocarotenoids, polyphenolics (flavonoids, anthocyanins catechins and phenolic acids) and organosulfur compounds (glucosinolates and allyl sulfides) as members of the main chemical classes of natural antioxidants. The influence of bioavailability enhancers on some of these antioxidants will also be discussed.

## 5. Bioavailability Studies of the Major Classes of Natural Antioxidants (1998–2013)

### 5.1. Carotenoids

**Figure 3:**
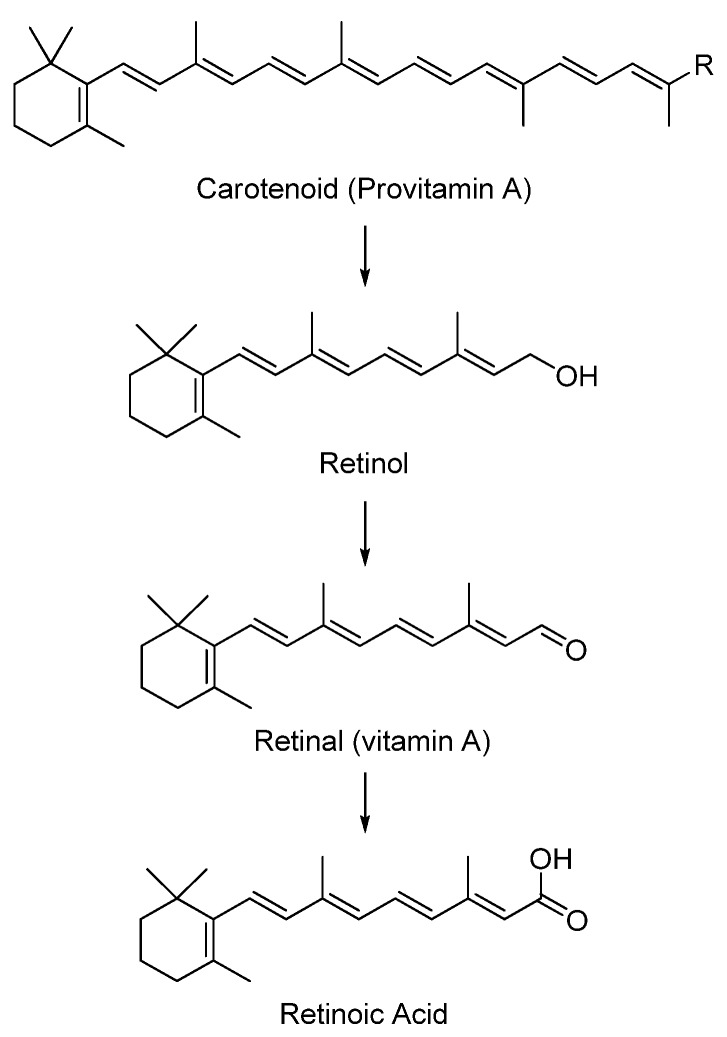
Biotransformation of structurally-relevant carotenoids to vitamin A and related analogs.

**Figure 4:**
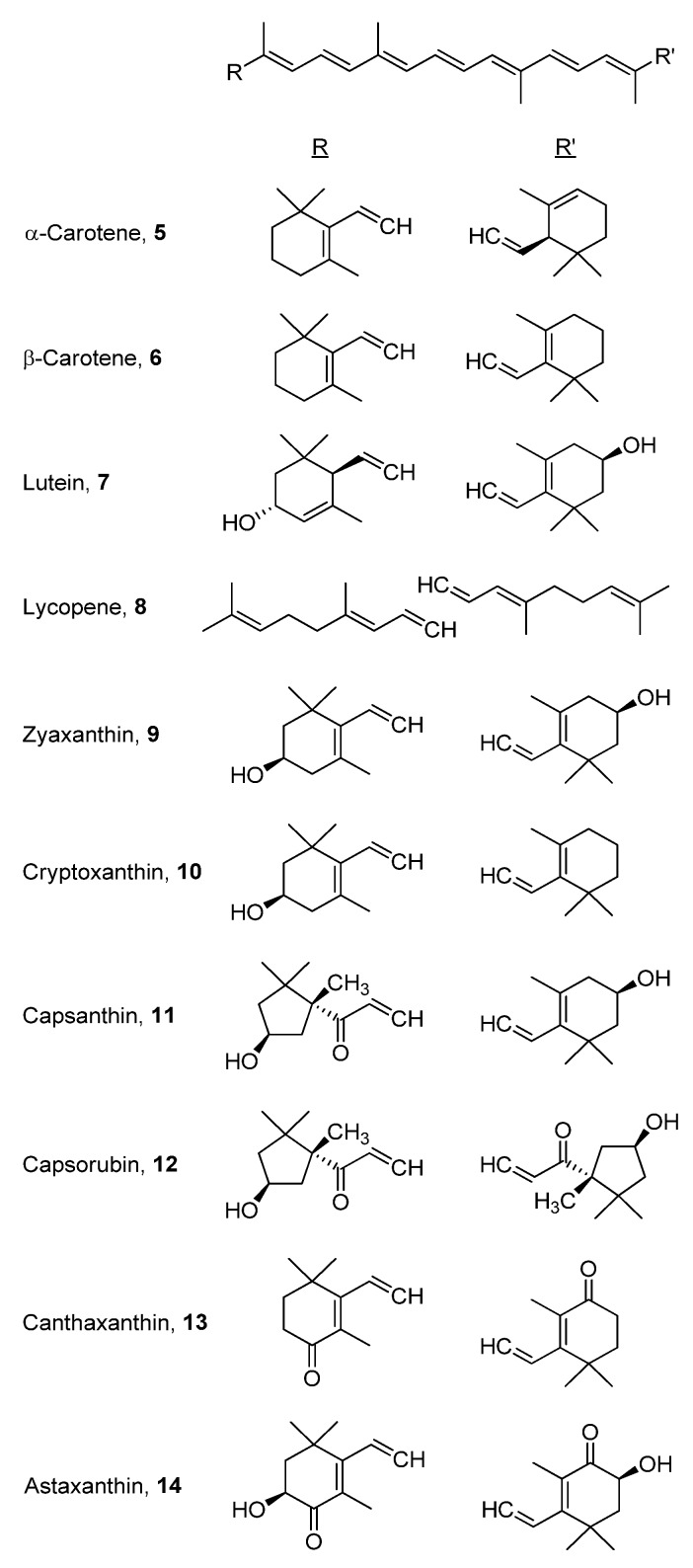
Food carotenoids whose bioavailability was recently investigated *in vitro* and/or *in vivo*.

Carotenoids, such as α-carotene (**5**), and apocarotenoids, such as crocetin (**3**), are a group of naturally occurring lipophilic pigments with extended conjugation that imparts yellow, orange or red colors to flowers and fruits [8]. The antioxidant activities of carotenoids are well established as scavengers of reactive oxygen species, as well as the ability of β-carotenes to act as provitamin A supplements in mammals [9]. The biotransformation of provitamin A to vitamin A analogs is shown in Figure 3. The health benefits and bioavailability of carotenoids have recently been described in a review by Fernandez-Garcia *et al*. [5]. In their review, the authors focused on food carotenoids, their levels in various constituents of the Mediterranean diet, digestion of fatty molecules, models for bioavailability evaluation, biotransformation of specific carotenoids to vitamin A (retinal) and the effect of dietary factors, such as fats and fibers, on the GI absorption of carotenoids. As discussed in the review, moderate amounts of dietary fat enhance the bioaccessibility (extent of release from the food matrix) of carotenoids for GI absorption, while high fatty content may deter their bioaccessibility. Formulation as emulsions in a hydrophilic matrix was also mentioned as an alternative approach towards enhancing the bioaccessibility/bioavailability of dietary carotenoids. The authors concluded by re-emphasizing the biological importance of carotenoids and the impact of various factors on their bioavailability. In another recent review, Borel discussed the effect of genetic variability on the bioavailability of carotenoids in humans [10]. Many other reviews were published that covered the bioavailability and/or bioaccessibility of the major food carotenoids, α- and β-carotene (**5** & **6**), lutein (**7**), lycopene (**8**), zeaxanthin (**9**) and cryptoxanthin (**10**) [9,11,12,13,14,15,16,17,18,19,20,21,22,23]. Others reviewed the effect of various factors on the bioavailability of carotenoids in general. The term “SLAMANGHI”, introduced by Castenmiller *et al*., adequately summarizes these main factors: species of absorption modifiers, molecular linkage, amount of carotene in meal, food matrix, absorption modifiers, personal nutrient status, genetic factors, host-related factors and interactions [24,25]. Recent investigations of some of the above-mentioned factors are summarized below with the chemical structures of the involved carotenoids shown in Figure 4. In many studies, bioaccessibility was often investigated alongside bioavailability.

The most investigated factors are probably food matrix and absorption modifiers, including methods of food preparation. The bioavailability of common food carotenoids (**5**–**10**) present in fresh vegetables/salads, such as carrots, parsley, spinach, tomato, red pepper, zucchini, and in fruits, such as bananas, bocaiuva pulp, mango and papaya, was investigated under different settings and was found to depend on the type and original concentration of the carotenoid(s) involved [26,27,28,29,30,31,32,33,34]. The less common carotenoids, capsanthin (**11**) and capsorubin (**12**), present in paprika oleoresin, had very low human bioavailability compared to common carotenoids also present in the resin [35], while the bioavailability of canthaxanthin (**13**), obtained from marine sources, was reduced when it was co-administered in rats with excess α-tocopherol (vitamin E, at 15–60 times the dose of canthaxanthin) [36]. The presence of oil as a food additive, such as in salad dressings or during cooking, generally acted as an absorption modifier, resulting in enhanced bioavailability of carotenoid compounds, as reflected in their degree of micellarization, passage into Caco-2 cells or plasma or the levels of retinol produced [37,38,39,40]. Co-administration of soy germ or testosterone had a similar enhancing effect on the bioavailability of carotenoids [41,42]. However, certain vegetable combinations, such as zucchini, red pepper and spinach, lead to the opposite effect when oil and/or fiber were added [30]. A recent study also demonstrated that different minerals may impact the bioavailability of spinach carotenoids. High sodium levels enhanced β-carotene and decreased lutein and zeaxanthin micellarization, respectively; while calcium and magnesium had a significant inhibitory effect [43]. Processed foods, mainly soups, tomato sauce/paste, broccoli, carrots and spinach, have been the subject of many bioavailability studies [39,44,45,46,47,48,49,50]. The results of these studies showed that incorporation of oil in cooking enhanced bioavailability, while heating during cooking decreased it. Furthermore, the bioavailability of many carotenoids was enhanced with frequent meals and higher dietary content. The bioavailability of pure individual carotenoids was also investigated [51,52,53]. In one study, the *in vitro* bioavailability of zeaxanthin in Caco-2 cells was shown to be equally high when the carotenoid was present in a free or esterified form [51]. In another study, it was found that oil had the same bioavailability-enhancing effect as with carotenoid-rich diets [52]. On the other hand, the hypocholesterolemic agent, ezetimibe, resulted in reduction of Caco-2 absorption when co-administered with β-carotene, but not lutein [53]. Another study demonstrated that the lipophilicity of pure β-carotene, lycopene, lutein and astaxanthin (**14**) improved their GI absorption, but not their adipose tissue uptake, after oral gavage in rats [54]. In addition, two studies focused on the effect of age on carotenoid bioavailability. Results from these studies showed that only the bioavailability of lycopene decreased with age [55,56].

**Figure 5:**
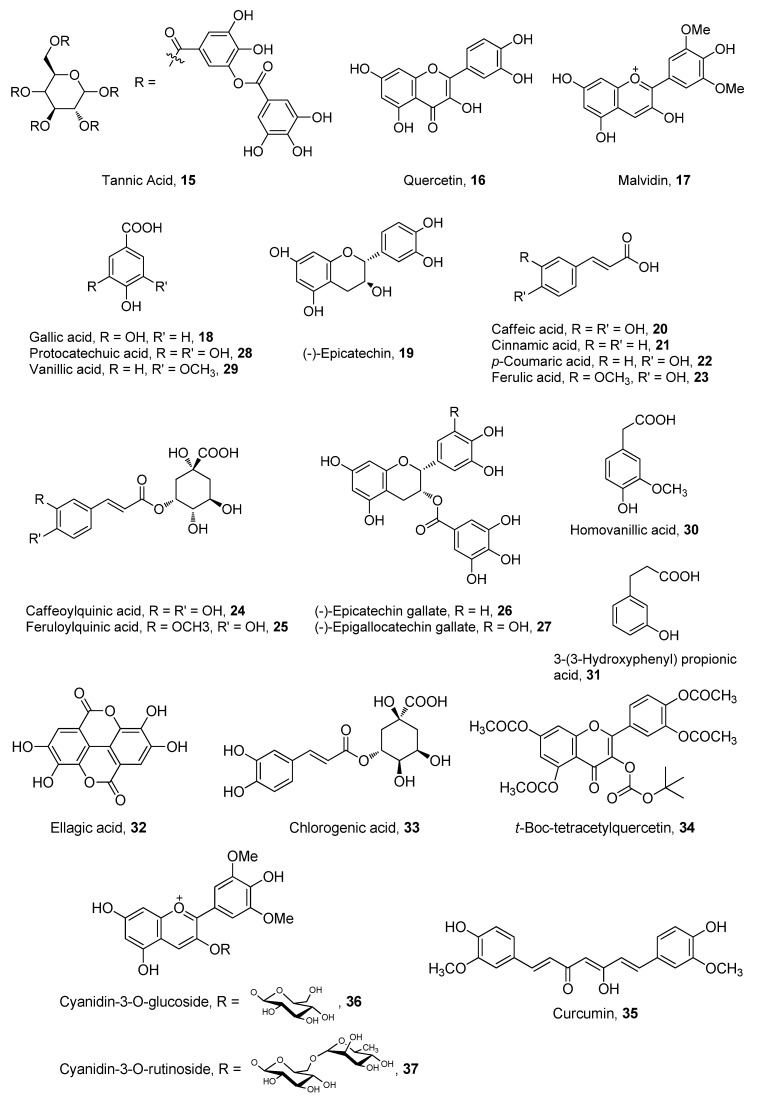
Polyphenolic compounds with recently investigated oral bioavailability.

### 5.2. Polyphenols

The base molecules of antioxidant plant phenols belong to such classes as flavonoids, anthocyanins, aromatic acids and quinones, characterized by the presence of polyhydroxy aromatic systems with one or more phenolic rings. They are also commonly known as polyphenolic compounds, which may exist as monomers, dimers or polymers of the base units, in an esterified or non-esterified form. As secondary metabolites, polyphenols have diverse functions in plants, the most noticeable of which is imparting characteristic colors, ranging from yellow to purple, to various organs, especially flowers, thus aiding in plant pollination by insects attracted to color [57]. Most plant polyphenols display significant antioxidant properties, mainly as free radical scavengers, which make them of interest for human therapeutics as potential chemopreventive agents and promoters of better health [57].

The bioavailability of polyphenolic compounds has been the subject of many recent reviews. Some reviews focused on polyphenols from individual plant sources, such as cocoa [58,59], lychee [60], tea [61,62,63], beer [64] and wine [65], while other reviews were more general [66,67,68,69,70]. There is agreement in many studies that the bioavailability of most polyphenols is not very high, due to such factors as poor absorption, instability, excessive metabolism or intestinal microbial transformation [71,72]. In spite of their poor bioavailability, there are indications that many polyphenols display activity at their observed low plasma concentrations [73]. Moreover, various approaches have been explored to enhance the bioavailability of polyphenols. Chemical derivatization, modified formulation (e.g., particle size and/or additives) and processing are the most common of such approaches. Polyphenols whose bioavailabilites have recently been investigated are shown in Figure 5, and the following is a summary of the most significant studies conducted in the past 15 years.

The bioavailabilites of catechin (**2**) and tannic acid (**15**) were evaluated *in vitro* in ligated rat small intestine segments. Although both compounds were absorbed by the intestinal wall (uptake: tannic acid 50%, catechin 30%), only catechin was shown to traverse the gut in low amounts (*ca*. 10%) [74]. The accumulation of quercetin (**16**) and malvidin (**17**) metabolites, quercetin-3-*O*-glucuronide and malvidin-3-*O*-glucoside, in rat brain after oral administration of red wine was detected and correlated with the generation of β-amyloid peptides [75]. Polyphenols and gallic acid (**18**) in grape seed were also monitored in the plasma of a rat model of Alzheimer’s disease after oral gavage of grape seed extract. The levels of gallic acid, catechin and epicatechin (EC, **19**) increased in plasma after repeated daily administration [76]. More than 30 polyphenol-derived metabolites were determined in human plasma after oral consumption of coffee. The presence of these metabolites demonstrated the bioavailability of caffeic (**20**), cinnamic (**21**), coumaric (**22**), ferulic (**23**), caffeoylquinic (**24**) and feruloylquinic (**25**) acids [77]. The effect of the particle size of a green tea product, Benifuuki variety, on the bioavailability of its polyphenols in rat models showed that 2 μm was optimum for the oral absorption of 19, epigallocatechin (EGC, **26**), epigallocatechin gallate (EGCG, **27**) and their *O*-methyl analogs [78]. In a study comparing the bioavailability of coffee and green tea polyphenols in humans, it was found that the phenolic acids of coffee, mainly ferulic and caffeic acids, were 1.7-fold more available than the green tea polyphenols, EC, EGC and EGCG [79]. The bioavailability of polyphenols present in a diet composed of bilberries, lingonberries, black currants and chokeberries (*ca.* 160 g/day) was determined in a human clinical trial. Quercetin, *p*-coumaric acid and 3-hydroxyphenylacetic acid were detected in both plasma and urine, while caffeic, protocatechuic (**28**), vanillic (**29**), homovanillic (**30**) and 3-(3-hydroxyphenyl)propionic (**31**) acids were only detected in plasma [80]. Raspberry anthocyanins and ellagitannins were also compared in healthy humans and in volunteers with ileostomy after oral consumption of 300 g of berries. Overall, the bioavailability of the mentioned polyphenols was very low, as indicated by urinary excretion, but the ileostomy volunteers showed 40% of anthocyanins and 23% of ellagitannins recovery in the ileal fluid. In addition, there were indications that ellagitannins were extensively metabolized to ellagic acid (**32**) in the stomach [81]. Various polyphenols were concurrently evaluated in a Caco-2 system and in a Sprague Dawley rat model of digestive stability and bioavailability. EC was found to be most stable, while EGC was least stable under digestive conditions. Polyphenol stability was enhanced by the addition of ascorbic acid and citric juices, which subsequently enhanced their bioavailability in both models [82]. In a pilot clinical trial, it was shown that milk protein had no significant effect on the bioavailability of catechin and EC present in cocoa butter after repeated co-administration at 8-h intervals [83]. The effect of milk and manufacturing procedures, such as spray drying, on the bioavailability of chlorogenic acid (**33**) present in coffee showed that approximately 40% of chlorogenic acid was bound to milk proteins. This binding was shown to decrease under *in vitro* digestion conditions, which may imply that it has no significant effect on bioavailability [84]. Ester derivatives of quercetin were synthesized in an attempt to reduce its phase II metabolism in GI epithelium. Transport of the quercetin derivatives was tested in MDCK-1, MDCK-2 and Caco-2 cells with varying degrees of absorption and metabolism. There were a few derivatives, such as Boc-protected tetraacetyl quercetin (**34**), that survived transport through MDCK and a few Caco-2 cell lines. According to the authors, these analogs may possess enhanced bioavailability when evaluated *in vivo* [85]. The enhancing effect of formulation on the bioavailability of polyphenols was demonstrated when an orally administered quercetin microemulsion significantly reduced the levels of interleukin 4 and 5 in a mouse model of airway allergic inflammation [86]. Curcumin (**35**)-loaded nanocapsules are another example in which formulation enhanced the bioavailability of a polyphenolic compound in a mouse melanoma model, whereby tumor volume was significantly reduced by the prepared formula. The formula was not orally administered, however, but was administered by intraperitoneal injection [87]. The bioavailability of polyphenols in rosemary, grape, citrus and marigold was correlated to the antioxidant effects of these plant extracts in sheep plasma after an acute oral dose of each. EC was one of the major polyphenols detected in plasma, especially after administration of grape extract [88]. Although they are significantly polar, nanomolar levels of two antioxidant cyanidin glycosides (**36** and **37**) were detected in rat plasma after 1 h of oral administration of 400 mg/kg of acai berry extract. The two glycosides were also detected in urine after 2 h from administration. However, there was no attempt to correlate bioavailability to *in vivo* antioxidant activity of the detected glycosides [89].

### 5.3. Organosulfur Compounds

**Figure 6:**
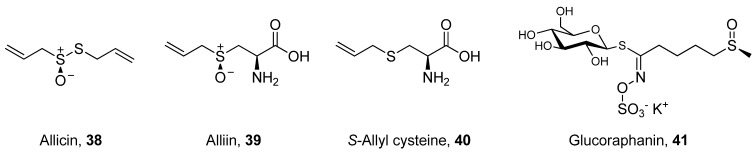
Organosulfur compounds with recently investigated oral bioavailability.

Glucosinolates are sulfur-containing plant secondary metabolites containing a thioketal-linked glucose molecule (*S*-glycosides). They usually exist in cruciferous plants and are hydrolyzed by specific enzymes (myrosinases) to release biologically active sulfurated aglycones, known as isothiocyanates [57]. Glucosinolates and their hydrolysis products exhibit direct and indirect antioxidant effects by scavenging harmful radicals and modulation of detoxification enzymes, such as glutathione *S*-transferase [90]. Thus, consumption of cruciferous plants, such as cabbage and broccoli, is believed to promote health and to reduce the risk of cancer development [91]. As shown in Figure 6, allicin (**38**), alliin (*S*-allylcysteine sulfoxide, **39**) and *S*-allylcysteine (SAC, **40**) constitute another class of organosulfur compounds, collectively known as cysteine sulfoxides, that are mainly present in garlic [57]. On hydrolysis, the lipid-soluble compounds (alliin and allicin) are converted to water-soluble products which are more stable, more bioavailable and exhibit antioxidant activity.

A relatively few number of reviews have been published over the past 15 years on the bioavailability of glucosinolates and organosulfur compounds [90,92,93,94]. Of these, the review by Holst and Williamson effectively summarizes the chemistry, biological activity and bioavailability of glucosinolates and their related analogs [90]. Similarly, not too many primary studies could be identified that were published during the surveyed period. One study may be considered an indicator of the bioavailability of Brussels sprout glucosinolates by demonstrating their effects on urine levels of aflatoxin-DNA adducts following oral administration of a hot water infusion of broccoli sprouts rich in glucoraphanin (**41**) [95]. Another study compared the levels of dithiocarbamates, glucosinolate metabolites, in urine of volunteers after consumption of cooked and uncooked broccoli sprouts. A recent study on the protective effect of SAC against aluminum-induced toxicity in rats demonstrated that SAC was of sufficient bioavailability to protect exposed rats after three days of oral administration. However, levels of SAC in biological fluids were not quantified after administration [96]. A computer-aided approach was utilized in another study to estimate certain pharmacokinetic properties of the organosulfur compounds of garlic. The most relevant properties predicted *in silico* were drug-plasma protein binding and the volume of distribution [97].

## 6. Conclusions

The main classes of plant-derived antioxidants for which most oral bioavailability studies have been published include the carotenoids, polyphenols and organosulfur compounds. Most of the published studies were focused on natural and processed foods, as well as herbal dietary supplements containing antioxidants belonging to the above-mentioned classes. Commonly reported factors affecting the bioavailability of natural antioxidants include chemical structure, processing methods, food additives and co-administered drugs, frequency of administration, as well as gender and genetic profile. Although the oral bioavailability of certain natural antioxidants, such as the polyphenols, is relatively low, there is evidence that they retain their biological activity at low plasma concentrations. Low bioavailability may also be enhanced via chemical modification and/or pharmaceutical formulation. *In vitro* models of bioavailability are useful in predicting/directing *in vivo* bioavailability, but clinical trials are the ultimate method for evaluating bioavailability, especially when conducted in tandem with pharmacological evaluation of an antioxidant in a specific disease condition. Because of the complex nature of many plant antioxidant-containing matrices, such as foods, dietary supplements and herbal extracts, it is difficult to obtain specific conclusions, but general bioavailability characteristics can be defined for each class, depending on the chemical nature of its antioxidant constituent(s). Consequently, bioavailability evaluation will continue to be tailored to, and conducted on, individual products as they emerge and depending on their composition and preparation method.
